# Parbendazole as a promising drug for inducing differentiation of acute myeloid leukemia cells with various subtypes

**DOI:** 10.1038/s42003-024-05811-8

**Published:** 2024-01-24

**Authors:** Hidemasa Matsuo, Aina Inagami, Yuri Ito, Nana Ito, Shinju Iyoda, Yutarou Harata, Moe Higashitani, Kota Shoji, Miu Tanaka, Mina Noura, Takashi Mikami, Itaru Kato, Junko Takita, Tatsutoshi Nakahata, Souichi Adachi

**Affiliations:** 1 Human Health Sciences, Graduate School of Medicine, Kyoto University, Kyoto, Japan; 2 Department of Integrated Health Sciences, Division of Cellular and Genetic Sciences, Nagoya University Graduate School of Medicine, Nagoya, Japan; 3 Department of Pediatrics, Graduate School of Medicine, Kyoto University, Kyoto, Japan; 4 Department of Fundamental Cell Technology, Center for iPS Cell Research and Application, Kyoto University, Kyoto, Japan

**Keywords:** Acute myeloid leukaemia, Drug development

## Abstract

Acute myeloid leukemia (AML) is a malignancy characterized by differentiation arrest of hematopoietic precursor cells. Differentiation therapy is effective for patients with acute promyelocytic leukemia; however, only a few effective differentiation therapies have been established for patients with other AML subtypes. In this study, seven benzimidazole anthelmintics were examined to determine the effects of differentiation on AML cells. The expression of monocyte markers (CD11b and CD14) was elevated after treatment with most benzimidazole anthelmintics. Among these drugs, parbendazole (PBZ) induced AML cell differentiation at low concentration. PBZ induced the monocyte marker expression, *KLF4/DPYSL2A* gene expression, and apoptosis for 21 AML cell lines with various subtypes and a primary AML sample. Finally, an in vivo analysis using an AML patient-derived xenograft mouse model showed a significant decrease in the chimerism level and prolonged survival in PBZ-treated mice. These findings could lead to a more effective differentiation therapy for AML.

## Abstract (web summary)

Low concentrations of the anthelmintic parbendazole (PBZ) induces monocytic differentiation in AML cells and PBZ could be a candidate for drug repositioning.

## Introduction

Acute myeloid leukemia (AML) is a malignancy characterized by differentiation arrest and uncontrolled proliferation of hematopoietic precursor cells^1^. Among the AML subtypes, the outcome of acute promyelocytic leukemia (APL) has been dramatically improved by the introduction of differentiating agents: all-trans retinoic acid (ATRA) and arsenic trioxide (ATO)^2,3^. Recently, isocitrate dehydrogenase (IDH) inhibitors are known to induce myeloid differentiation in patients with AML with *IDH1/2* mutations^4,5^. However, these therapies apply only to a small number of AML patients, and only a few effective differentiation therapies have been established for patients with other AML subtypes.

Krüppel-like factor 4 (KLF4) is a transcription factor that regulates diverse cellular processes, such as cell growth, proliferation, and differentiation^6^. KLF4 repression is associated with leukemogenesis, and reactivated KLF4 in AML cells has been shown to function as a potent terminal differentiation mediator in the monocytes^7,8^. Our previous study revealed that KLF4 directly transactivates the expression of dihydropyrimidinase-like 2A (DPYSL2A, also known as CRMP2, a member of the collapsin response mediator protein family) and that the KLF4-DPYSL2A axis serves as a promising target for differentiation therapy^9^. Moreover, a drug screening identified albendazole (ABZ) as a candidate drug to induce AML cell differentiation to monocytes by stimulating the KLF4-DPYSL2A axis^10^. ABZ is a Food and Drug Administration (FDA)-approved broad-spectrum anthelmintic with low toxicity and is widely used in humans and animals^11^. Recently, ABZ has been reported to induce antitumor activities in various cancer types^12–14^. Moreover, several studies demonstrated that other benzimidazole anthelmintics (fenbendazole, flubendazole, and mebendazole) also induce anti-leukemic activities^15–17^. However, whether the anti-leukemic activity varies by benzimidazole anthelmintics and whether these drugs induce differentiation for AML cells with various subtypes are unclear.

Here, we demonstrate that most benzimidazole anthelmintics with chemical structures similar to ABZ can induce AML cell differentiation to monocytes. Among these drugs, parbendazole (PBZ) induced differentiation at lower concentrations for AML cells with various subtypes. These findings could lead to providing a more effective AML differentiation therapy through drug repositioning.

## Results

### Induction activity of AML cell differentiation by benzimidazole anthelmintics

**Fig. 1:**
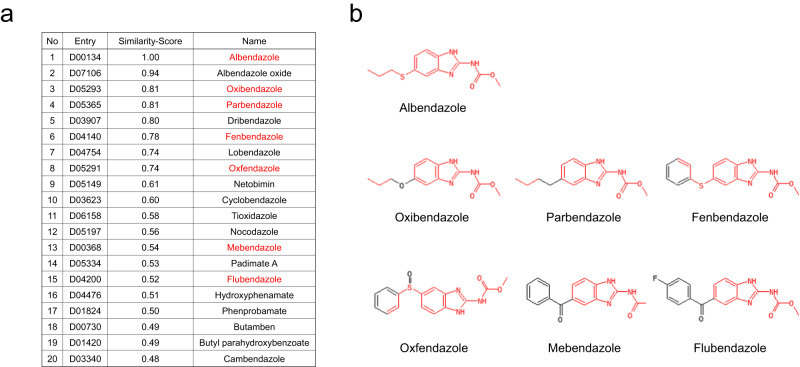
Determination of seven benzimidazole anthelmintics examined in this study. **a** The top 20 drugs with high similarity scores with ABZ were identified using SIMCOMP, a similar compound search tool. Of these, seven benzimidazole anthelmintics including ABZ were selected for further analysis (highlighted in red). **b** The chemical structures of each drug are shown. The drugs had different substituents at the 5th position of the benzimidazole nucleus. The shared structure with ABZ is highlighted in red.

To select compounds with chemical similarity to ABZ, SIMCOMP, a similar compound search tool, was used. The top 20 drugs with a high similarity score to ABZ were identified (Fig. 1a). Of these, seven benzimidazole anthelmintics including ABZ were selected for further analysis. The chemical structures were similar, and the drugs had different substituents at the 5th position of the benzimidazole nucleus (Fig. 1b).

**Fig. 2:**
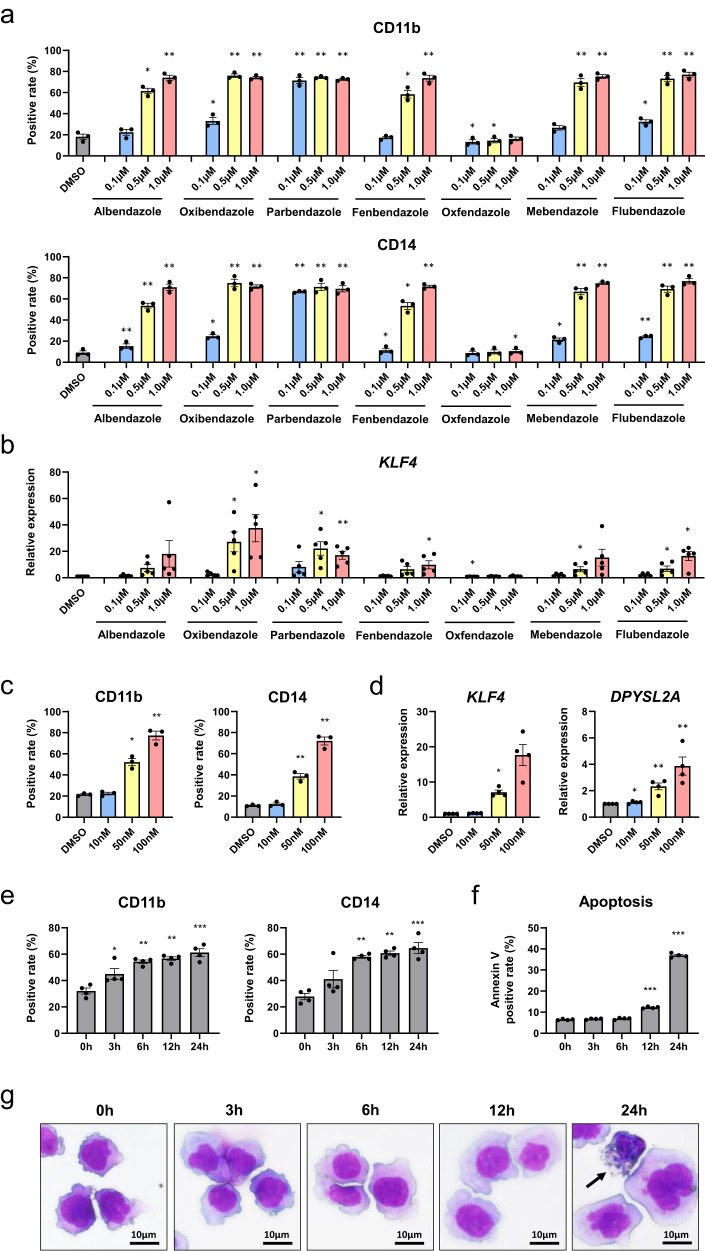
Induction activity of AML cell differentiation by benzimidazole anthelmintics. **a** Induction activity of AML cell differentiation by seven benzimidazole anthelmintics was analyzed. THP-1 cells were treated with each drug for 48 h, and the expression of monocyte markers (CD11b and CD14) was analyzed by FCM. **b** THP-1 cells were treated with each drug for 48 h, and *KLF4* expression was examined by RT-qPCR. **c** The induction activity of AML cell differentiation by PBZ at lower concentrations was examined. THP-1 cells were treated with PBZ (10 nM, 50 nM, or 100 nM) for 48 h, and the expression of monocyte markers (CD11b and CD14) was analyzed by FCM. **d** THP-1 cells were treated with PBZ (10 nM, 50 nM, or 100 nM) for 48 h, and *KLF4/DPYSL2A* expression was examined by RT-qPCR. **e** THP-1 cells were exposed to PBZ (100 nM) for 0, 3, 6, 12, and 24 h, and monocyte marker expression (CD11b and CD14) was analyzed by FCM. f The percentage of Annexin V-positive apoptotic cells in THP-1 cells treated with PBZ (100 nM) for 0, 3, 6, 12, and 24 h was determined by FCM. **g** Morphological changes in THP-1 cells treated with PBZ (100 nM) for 0, 3, 6, 12, and 24 h were observed. Morphological alterations, such as decreased nuclear-to-cytoplasmic ratio and indented nucleus, were noted after 3 h of PBZ treatment. Apoptotic cells (black arrow) were observed after 24 h of treatment. Data are presented as the mean ± SEM. *P < 0.05, **P < 0.01, ***P < 0.001.

First, the induction activity of AML cell differentiation by seven benzimidazole anthelmintics was assessed using an AML cell line: THP-1. Flow cytometry (FCM) was used to analyze the expression of monocyte markers (CD11b and CD14). The ABZ treatment increased the CD11b and CD14 expression in a dose-dependent manner (Fig. 2a). Similar results were obtained in samples treated with oxibendazole, fenbendazole, mebendazole, or flubendazole. Only oxfendazole did not successfully induce the elevation of monocyte markers in THP-1 cells. Conversely, the sample treated with PBZ showed increased CD11b and CD14 expressions at a low concentration (0.1 µM). To analyze the differentiation mechanism of AML cells to monocytes, *KLF4* expression was examined by real-time quantitative polymerase chain reaction (RT-qPCR). As a result, *KLF4* expression was elevated by treatment with benzimidazole anthelmintics, except oxfendazole (Fig. 2b). These data were compatible with the FCM result. Furthermore, the induction activity of AML cell differentiation by PBZ at lower concentrations was examined. PBZ treatment (≧50 nM) significantly increased CD11b and CD14 expressions (Fig. 2c) and *KLF4/DPYSL2A* expression (Fig. 2d). The effects of PBZ (0.1 µM) on AML cell differentiation and apoptosis within 24 h were investigated using FCM and morphological observation. The results showed that monocyte markers (CD11b and CD14) increased significantly within 6 h, while the positive rate of Annexin V (indicative of apoptotic cells) rose after 12 h (Fig. 2e, f). These findings were consistent with the results from morphological observation (Fig. 2g). Therefore, PBZ is suggested to induce AML cell differentiation, with the differentiated cells rapidly undergoing apoptosis. These data suggest that most benzimidazole anthelmintics can effectively induce AML cell differentiation and that PBZ may be effective at lower concentrations compared with other drugs.

### Effects of benzimidazole anthelmintics on AML cell viability

**Fig. 3:**
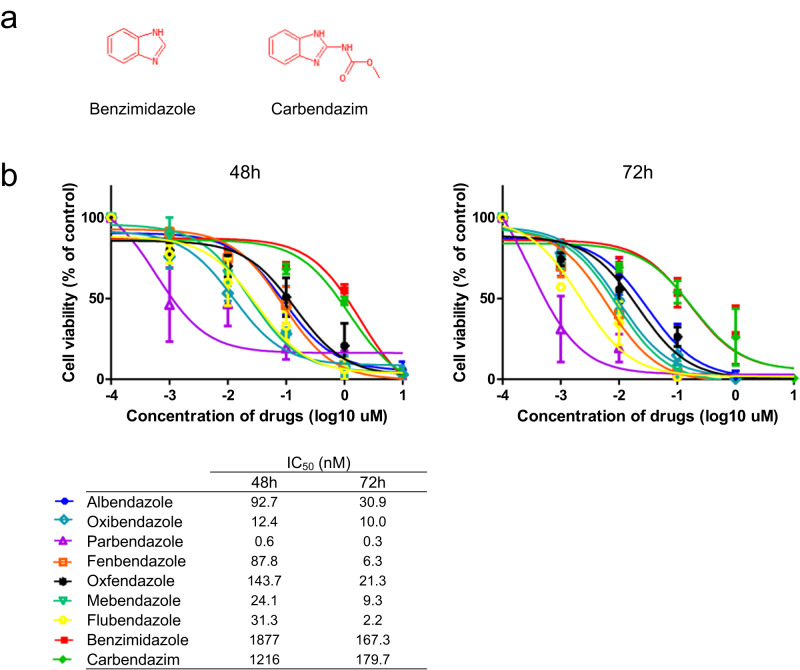
Effects of benzimidazole anthelmintics on AML cell viability. **a** The chemical structures of the scaffold compounds (benzimidazole and carbendazim). **b** The effects of benzimidazole anthelmintics on AML cell viability were examined by IC_50_ analysis using THP-1 cells. Among the examined drugs, PBZ showed the lowest IC_50_ value at both 48 h and 72 h. Conversely, the scaffold compounds showed high IC_50_ values.

Next, the effects of benzimidazole anthelmintics on AML cell viability were examined by IC_50_ analysis using THP-1 cells. In addition to the seven benzimidazole anthelmintics, scaffold compounds (benzimidazole and carbendazim) were examined to identify the anti-leukemic properties of these scaffold compounds lacking substituents at the 5th position of the benzimidazole nucleus. Their chemical structures are shown in Fig. 3a. Among the examined drugs, PBZ showed the lowest IC_50_ value at both 48 h and 72 h (Fig. 3b). In contrast, the scaffold compounds (benzimidazole and carbendazim) showed high IC_50_ values, suggesting the need for high concentrations to interfere with AML cell viability. These data suggest that PBZ has anti-leukemic properties at lower concentrations than other drugs. The substituents at the 5th position of the benzimidazole nucleus may be important for the activity. To determine whether the efficacy of PBZ is specific to AML cell lines, we assessed the IC_50_ for other cancer cell lines, including pancreatic cancer (AsPC-1), colon cancer (SW620), acute lymphoblastic leukemia (Jurkat), and Burkitt lymphoma (Daudi). The results revealed that PBZ’s IC_50_ for most cell types is at the nanomolar level, with values lower than those of ABZ (Supplementary Fig. 1). Consequently, PBZ may prove effective against various cancer cell types.

### Effects of PBZ on the differentiation of AML cells with various subtypes

**Fig. 4:**
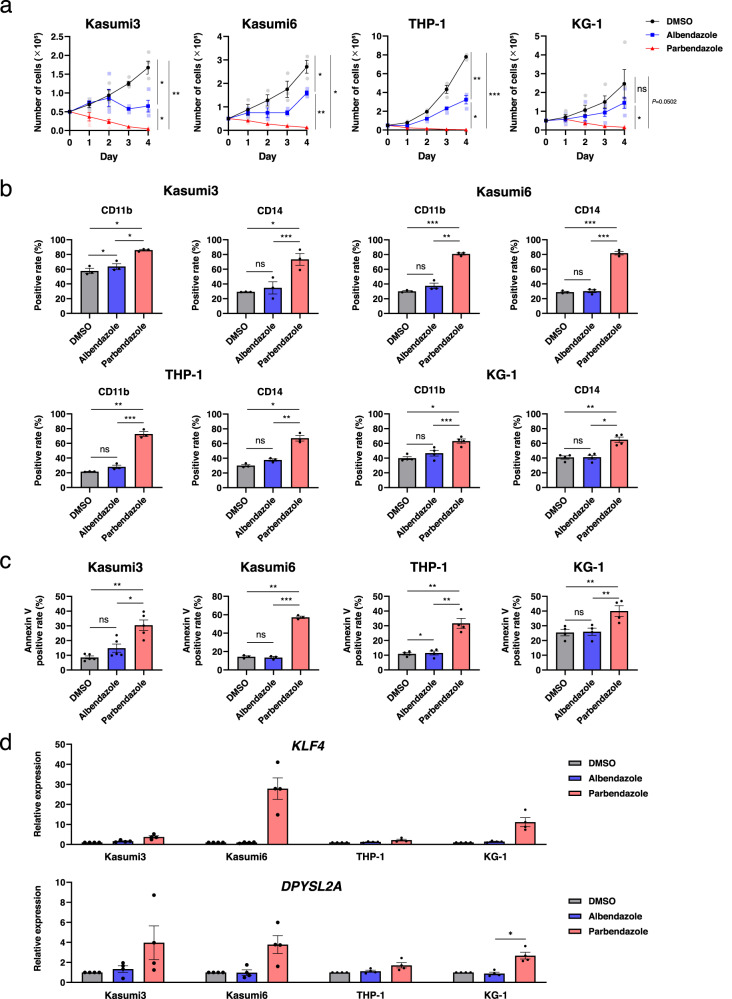
Effects of PBZ on the differentiation of AML cells with various subtypes. **a** AML cell lines (Kasumi3, Kasumi6, THP-1, and KG-1) were treated with DMSO, ABZ (100 nM), or PBZ (100 nM), and the number of cells was counted for 4 days. The full results of all 21 AML cell lines are shown in Supplementary Fig. 1. AML cell lines were treated with DMSO, ABZ (100 nM), or PBZ (100 nM) for 48 h, and the expression of monocyte markers (CD11b and CD14) (b), the percentage of Annexin V positive apoptotic cells (**c**), and *KLF4/DPYSL2A* expression (**d**) were examined. The full results of all 21 AML cell lines are shown in Supplementary Figs. 2–4. Data are presented as the mean ± SEM. *P < 0.05, **P < 0.01, ***P < 0.001. ns, not significant.

We next examined whether PBZ has superior activity of the effects in AML cell differentiation with various subtypes, compared with ABZ. Overall, 21 AML cell lines with various French–American–British (FAB) classifications and genetic abnormalities were used (Supplementary Table 1). Results of four representative AML cell lines (Kasumi3, Kasumi6, THP-1, and KG-1) are shown in Fig. 4, and those of all 21 AML cell lines are shown in Supplementary Figs. 2–5. AML cell lines were treated with DMSO, ABZ, or PBZ. The drugs were used at a 100 nM concentration because 100 nM ABZ did not sufficiently induce THP-1 cell differentiation, whereas 100 nM PBZ was sufficient (Fig. 2a). In most AML cell lines, cell proliferation was inhibited by both ABZ and PBZ; however, a marked inhibition was observed in PBZ-treated samples, irrespective of the FAB classifications and genetic abnormalities of AML cells (Fig. 4a, Supplementary Fig. 2). PBZ treatment significantly increased CD11b and CD14 expressions and induced apoptosis in AML cell lines (Fig. 4b, c, Supplementary Figs. 3 and 4). Concordantly, PBZ treatment also resulted in increased *KLF4* expression in AML cell lines (Fig. 4d, Supplementary Fig. 5). Furthermore, the effects of PBZ on normal bone marrow cells were examined. The colony-forming capacity of the c-kit+ immature bone marrow cells from wild-type mice did not respond to PBZ treatment up to 100 nM (Supplementary Fig. 6). Additionally, human CD34+ cord blood from two donors demonstrated minimal apoptosis increase after 48 h incubation with 100 nM PBZ (Supplementary Fig. 7). These data suggest that PBZ has superior effects for inducing AML cell differentiation with various subtypes, and the effect is limited in normal bone marrow cells.

### Effects of PBZ on AML-patient delived xenografted (PDX) cells in vitro and in vivo

**Fig. 5:**
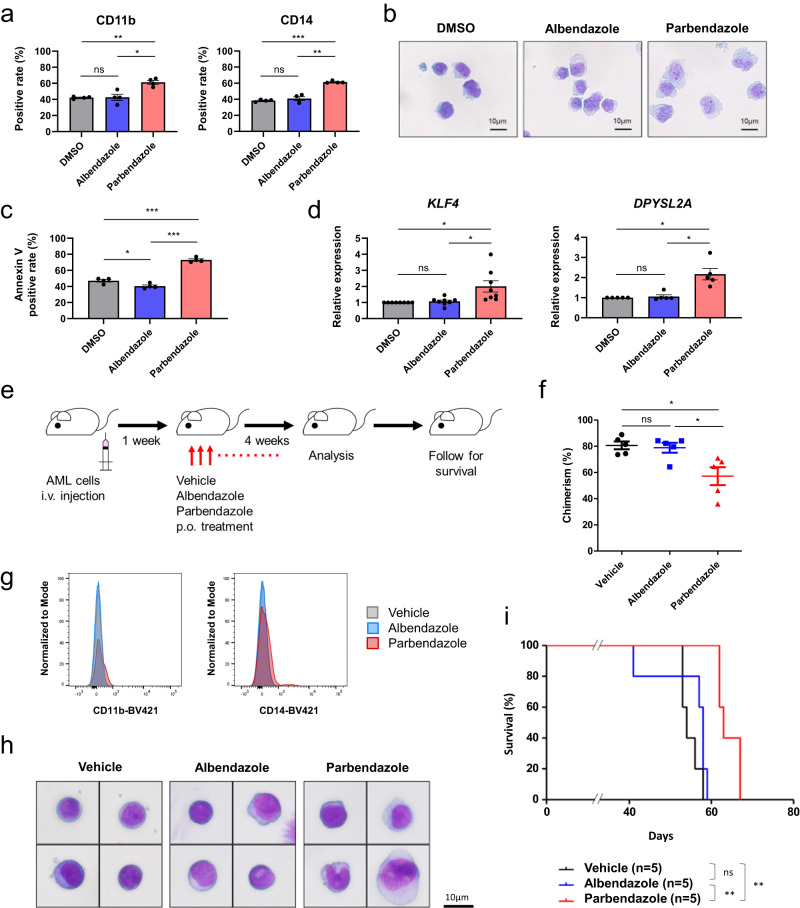
PBZ has anti-leukemia effects on AML-PDX cells in vitro and in vivo. AML-PDX cells were treated with DMSO, ABZ (100 nM), or PBZ (100 nM) for 48 h, and the expressions of monocyte markers (a), morphology (**b**), apoptosis (**c**), and *KLF4/DPYSL2A* mRNA expression (**d**) were examined in vitro. (**e**). Schematic illustration of an in vivo study. NOG mice were injected with AML-PDX cells. One week later, they were administered a control vehicle, ABZ, or PBZ (100 mg/kg body weight, orally, once daily). Five weeks after AML-PDX cell transplantation, the mice were assessed for chimerism and cell differentiation. (f) Chimerism percentages were compared. (g) Expression of monocyte markers (CD11b and CD14) was compared using FCM on AML-PDX cells sorted from mouse bone marrow. **h** Morphological changes in AML-PDX cells from mouse bone marrow were examined. **i** Survival rates of mice treated with DMSO, ABZ, or PBZ were compared. Data are presented as mean ± SEM. *P < 0.05, **P < 0.01, ***P < 0.001. ns, not significant.

Finally, we examined whether PBZ has anti-leukemic properties for patient-derived AML cells. Leukemia cells derived from a patient with *KMT2A*-rearranged AML were expanded in NOD/Shi-scid, IL-2RγKO (NOG) mice as AML-PDX cells and used for further examinations. AML-PDX cells were treated with DMSO, ABZ (100 nM), or PBZ (100 nM) for 48 h, and the expressions of monocyte markers, morphology, apoptosis, and *KLF4/DPYSL2A* mRNA expression were examined in vitro. PBZ treatment significantly induced AML cell differentiation as evidenced by increased expression of monocyte markers (CD11b and CD14) (Fig. 5a). PBZ treatment showed the following morphological changes in AML-PDX cells: decreased nuclear-to-cytoplasmic ratio, indented nucleus, reduced cytoplasmic basophilia, and occurrence of cytoplasmic vacuoles (Fig. 5b). These morphological characteristics were compatible with those of monocytes. PBZ treatment also effectively induced apoptosis (Fig. 5c) and elevated the *KLF4/DPYSL2A* expression in AML-PDX cells (Fig. 5d).

For in vivo analysis, we initially assessed the impact of PBZ on blood cell counts and body weight in C57BL/6 J mice. The results revealed no significant alterations in blood cell counts or body weight throughout the 28-day study at concentrations up to 100 mg/kg (Supplementary Fig. 8). Subsequently, we investigated the in vivo anti-leukemic activity using a xenograft model. NOG mice received injections of AML-PDX cells and were treated with either a control vehicle, ABZ, or PBZ (100 mg/kg body weight, administered orally) (Fig. 5e). The chimerism percentage significantly decreased in PBZ-treated mice compared to DMSO/ABZ-treated mice (Fig. 5f). AML-PDX cells sorted from mouse bone marrow exhibited partial differentiation, as evidenced by increased expression of monocyte markers (CD11b and CD14) and morphological changes (Fig. 5g, h). Moreover, PBZ-treated mice demonstrated an extended survival time compared to the other groups (Fig. 5i). These results indicate that PBZ exerts anti-leukemic effects on AML-PDX cells, both in vitro and in vivo.

## Discussion

This study analyzed the effects of benzimidazole anthelmintics on AML cell differentiation. Most benzimidazole anthelmintics induced AML cell differentiation to monocytes. ABZ, fenbendazole, flubendazole, and mebendazole have been reported as candidate drugs harboring anti-leukemic activity, which is consistent with our data^10,15–17^. Among the examined drugs, PBZ induced differentiation at lower concentrations of AML cells. Studies on repurposing benzimidazole anthelmintics for cancer treatment have gradually gained popularity^18^; however, only a few studies have shown a difference in the anti-cancer activity among benzimidazole anthelmintics. Our study revealed that, in addition to AML cells, PBZ exhibits superior efficacy against various cancer cell lines.

PBZ, an FDA-approved drug, is currently used to treat parasitic infections in animals, not humans^19,20^. The drug has been reported as a candidate drug for repositioning various cancers, including pancreatic cancer and head and neck squamous cell carcinoma^19,21,22^. The chemical structure of PBZ and other benzimidazole anthelmintics differ in terms of substituents at the 5th position of the benzimidazole nucleus. Moreover, the scaffold compounds (benzimidazole and carbendazim) showed high IC_50_ values. These data suggest that scaffold compounds of benzimidazole anthelmintics cannot sufficiently induce AML cell differentiation and that the 5^th^ position of the benzimidazole nucleus is associated with efficacy.

PBZ effectively induced differentiation at lower concentrations for AML cell lines with various FAB subtypes and genetic abnormalities. As a possible mechanism for inducing AML cells to monocytes by PBZ, we showed an elevated *KLF4/DPYSL2A* expression by PBZ in AML cells. PBZ increased *KLF4* expression in a concentration-dependent manner (Fig. 2d). However, the concentration dependence was lost at higher concentrations (Fig. 2b), possibly due to PBZ’s high efficacy at low concentrations, leading to significantly reduced cell viability at high concentrations, such as 1 µM. In a previous study, ABZ was identified by drug screening as a promising drug that can differentiate AML cells by stimulating the *KLF4/DPYSL2A* axis^10^; therefore, PBZ, and ABZ may share the same pathway during the exertion of anti-leukemic activity. Despite PBZ’s broad impact on differentiating various AML cells, its effect on normal bone marrow cells was limited, possibly because *DPYSL2A* expression is generally higher in normal hematopoietic stem cells compared to AML cells^9^. Benzimidazole derivatives act as anthelmintics by inhibiting microtubule polymerization^23^; however, vincristine, a representative microtubule inhibitor, did not induce monocytic markers^10^. Therefore, the induction of AML cell differentiation by stimulating the *KLF4/DPYSL2A* axis may be independent of the inhibition of microtubule polymerization. The identification of the molecular target and the detailed mechanisms of PBZ efficacy may also lead to the development of more selective drugs.

Moreover, PBZ exhibited in vivo anti-leukemic effects on AML-PDX cells. However, its administration only achieved partial reduction in chimerism, induced limited differentiation of AML cells, and marginally extended mouse survival. This may be attributed to the aggressiveness and heterogeneity of the transplanted AML-PDX cells. Further research is required to understand PBZ resistance mechanisms and to explore strategies enhancing PBZ’s efficacy, including optimized administration methods and potential drug combinations. At present, the differentiation therapy of AML applies only to APL and AML with *IDH1/2* mutations^2–5^. PBZ may be a promising lead compound for the induction therapy of the other AML subtypes.

## Methods

### Cell lines

HYT-1, THP-1, KG-1a, and M-MOK cells were purchased from RIKEN BioResource Research Center, Japan. Kasumi1, Kasumi3, Kasumi6, SKNO1, HL-60, NOMO-1, Jurkat, and Daudi cells were obtained from the Japanese Collection of Research Bioresources (Japan). KO52, ML-2, OCI-AML2, OCI-AML3, MOLM13, MV4-11, U937, and HEL cells were purchased from Deutsche Sammlung von Mikroorganismen und Zellkulturen GmbH (DSMZ, Germany). AsPC-1 and SW620 cells were purchased from the American Type Culture Collection (ATCC, USA). ATRA-resistant APL-derived NB4 and UF-1 cells were kindly provided by Dr. Y. Ikeda (Keio University School of Medicine, Japan). AML-derived ME-1 cells were a gift from Dr. PP Liu (National Human Genome Research Institute, National Institutes of Health, USA). Kasumi1 cells were cultured in Roswell Park Memorial Institute (RPMI) 1640 medium containing 20% fetal bovine serum (FBS) and 1% penicillin-streptomycin-glutamine (PSG). HYT-1 cells were cultured in the RPMI 1640 medium containing 10% FBS, 1% PSG, and 10 ng/ml human G-CSF. Kasumi6 cells were cultured in the RPMI 1640 medium containing 20% FBS, 1% PSG, and 2 ng/ml human GM-CSF. M-MOK cells were cultured in the RPMI 1640 medium containing 10% FBS, 1% PSG, and 10 ng/ml human GM-CSF. SW620 cells were cultured using Dulbecco’s Modified Eagle Medium containing 10% FBS and penicillin-streptomycin. Other cell lines were cultured in RPMI 1640 medium containing 10% FBS and 1% PSG. All cell lines were cultured at 5% CO_2_ and 37 °C.

### Reagents

SIMCOMP^24^, a similar compound search tool, was used to identify 20 drugs with structures similar to that of ABZ. Of these, seven benzimidazole anthelmintics including ABZ were selected for examination. ABZ, oxibendazole, PBZ, fenbendazole, oxfendazole, mebendazole, and flubendazole were purchased from MedChemExpress, USA. Scaffold compounds (benzimidazole and carbendazim) were used as control. Benzimidazole was purchased from Tokyo Chemical Industry Co., Ltd. Carbendazim was synthesized from Mcule, USA.

### RT-qPCR

Total RNA was isolated using the RNeasy mini kit (Qiagen, USA) and reverse-transcribed using ReverTraAce^®^ qPCR RT Master Mix (TOYOBO, Japan) to generate cDNA. For cell lines with low DPYSL2A expression, reverse transcription was performed using SuperScript™ RT (Invitrogen) to increase the amount of template. qRT-PCR was performed using the Step One Plus^TM^ Real-Time PCR System (Applied Biosystems, USA), and detection was performed using TB Green^®^ Premix Ex Taq^TM^ II (Tli RNaseH Plus) (TaKaRa). The results were interpreted based on the 2^-ΔΔCt^ method and corrected for glyceraldehyde-3-phosphate dehydrogenase (GAPDH), and relative quantification was performed with DMSO of 1, the control. The results are presented as the mean ± standard error of the mean (SEM) of the values obtained from at least three independent experiments. The primers used for qRT-PCR are listed in Supplementary Table 2.

### IC_50_ evaluation

For the cell viability assay, cells were seeded onto 96-well plates at a density of 1 × 10^4^–10^5^ cells/well and treated with each compound at different concentrations for 48 and 72 hours. Cell viability was assessed by the WST assay using Cell Count Reagent SF (Nacalai Tesque, Inc., Japan) and an Infinite^®^ 200 PRO multimode reader (TECAN, Switzerland). Percentage inhibition curves were drawn, and IC_50_ values of the compounds were calculated using GraphPad Prism 5 Software (MDF, Japan).

### Cell proliferation assay

AML cells were seeded onto 6-well plates at a density of 1 × 10^5^ cells/well. Cell counts were measured using Countess™ II Automated Cell Counter (Thermo Fisher Scientific, USA) for 5 days. The results are presented as the mean ± SEM of the values obtained from at least three independent experiments.

### FCM

Monocytic AML cell differentiation was assessed using brilliant violet 421 (BV421)-labeled anti-human CD11b (301324; BioLegend, USA) and CD14 (325628; BioLegend, USA) antibodies. The living cell population was gated in a forward scatter/side scatter dot plot; then, CD11b, and CD14 expressions were analyzed. Apoptotic cells were determined using the APC Annexin V Apoptosis Detection Kit with PI (640932; BioLegend, USA) or 7-AAD (420403; BioLegend, USA). For chimerism analysis, bone marrow cells were collected by bone marrow aspiration and reacted with PE-labeled anti-mouse CD45 (561087; BD Biosciences, USA) and FITC-labeled anti-human CD45 (368508; BioLegend, USA) antibodies on ice for 30 min in the dark. The chimerism percentage in the bone marrow of mice was evaluated using the fact that mouse-derived cells are mCD45-positive and leukemia cells are hCD45-positive. The gating strategy is provided in the Supplementary Fig. 9. Measurements/cell sorting were performed using a BD FACS Canto™ II or BD FACS Aria™ II (BD Biosciences, USA). The Flow Jo software (BD Biosciences, USA) was used for data analysis. To determine the CD11b/CD14 positivity rate for each cells, a positivity threshold was determined using the histogram of the DMSO-treated samples (control). The CD11b/CD14 positivity rate in ABZ/PBZ treated samples was then examined using the threshold. The results are presented as the mean ± SEM of the values obtained from at least three independent experiments.

### Morphology

Cells were spun onto slides using the StatSpin Cytofuge 2 Cytocentrifuge (Beckman Coulter, USA). Then, Diff–Quik staining (modified Giemsa staining) was performed on each slide.

### Mice

C57BL/6 J mice were purchased from CLEA Japan, Inc., and NOG mice were obtained from the Central Institute for Experimental Animals, Inc. All procedures performed in this study were approved by the Kyoto University Animal Experimentation Committee (Permit Number: Med Kyo 22530).

### Colony-forming assay

C-kit+ primary bone marrow cells of the mouse were isolated from C57BL/6 J mice using APC anti-mouse c-kit antibody (105812; BioLegend, USA) and BD FACS Aria™ II (BD Biosciences, USA). Isolated cells were plated onto MethoCult M3434 Classic media (StemCell Technologies, Canada), and the number of colonies formed was counted 10 days post-treatment.

### Human CD34+ cord blood

Human CD34+ cord blood cells from two donors was purchased from STEMCELL Technologies Inc., USA (CAT# 70008.2, donor #1: male, donor #2: female). The cells were cultured in MethoCult H4034 Optimum (ST-04044, STEMCELL Technologies Inc., USA).

### Mice toxicity test

C57BL/6 J mice (female, 14 weeks old) were randomly divided into four groups, each consisting of three mice. They were administered either the control vehicle or PBZ (at doses of 10, 50, and 100 mg/kg) via daily oral administration. The maximum dose of 100 mg/kg was selected based on a previous study^22^. On days 14 and 28, peripheral blood cell counts were obtained using Celltac α (Nihon Kohden, Tokyo, Japan), and the mice’s body weights were recorded.

### Patient specimen

Primary AML cells were obtained from the bone marrow of a pediatric AML patient at Kyoto University Hospital after an institutional review board approval (Permit Number: G1030) and providing informed consent. The patient was initially diagnosed as *KMT2A-AFF1*-positive B-cell precursor acute lymphoblastic leukemia; however, the lineage was switched to AML (FAB: M5) during treatment. The bone marrow cells at the AML stage were obtained and expanded using NOG mice as AML-PDX cells for further analysis. This study was conducted following the Declaration of Helsinki.

### AML mice model

AML-PDX cells, prepared at 1.0 × 10^6^ cells per 200 μL of MEMα (Thermo Fisher Scientific), were intravenously injected into 15 NOG mice (male, 28 weeks old). One week post-transplantation, the mice were divided into three random groups (five mice per group). Treatment began with ABZ or PBZ (100 mg/kg body weight, daily oral administration) or an equivalent amount of control vehicle. Five weeks after AML-PDX cell transplantation, the mice were evaluated for chimerism and cell differentiation. Subsequently, mice survival was monitored without further treatment. Kaplan–Meier curves were drawn using GraphPad Prism 5/10 Software (MDF, Tokyo, Japan).

### Statistics and reproducibility

Continuous variables were compared using the Student’s *t*-test or Mann–Whitney *U*-test. Results are presented as the mean ± SEM obtained from at least three independent experiments. A *P*-value of <0.05 was considered statistically significant. Statistical significance was defined as follows: *P < 0.05, **P < 0.01, or ***P < 0.001.

### Reporting summary

Further information on research design is available in the Nature Portfolio Reporting Summary linked to this article.

### Supplementary information

Supplementary Information

Description of Additional Supplementary Files

Supplementary Data 1

Reporting Summary

## Data Availability

All source data for the graphs and charts are present in Supplementary Data 1. Additional data are available from the corresponding author on reasonable request.
