# Supplementary material for: Parbendazole as a promising drug for inducing differentiation of acute myeloid leukemia cells with various subtypes
Source: Commun Biol. 2024 Jan 24;7:123. doi: 10.1038/s42003-024-05811-8 (PMC10808455; doi:10.1038/s42003-024-05811-8)
Supplement: Supplementary file 1 — Supplementary Information [file 42003_2024_5811_MOESM1_ESM.pdf]

Supplemental files

Parbendazole as a promising drug for inducing differentiation of acute myeloid leukemia cells with various subtypes

Hidemasa Matsuo, Aina Inagami, Yuri Ito, Nana Ito, Shinju Iyoda, Yutarou Harata, Moe Higashitani, Kota Shoji, Miu Tanaka, Mina Noura, Takashi Mikami, Itaru Kato, Junko Takita, Tatsutoshi Nakahata, and Souichi Adachi

Supplementary Table 1

FAB subtypes and major genetic abnormalities of cell lines used in this study.

Cell line	FAB subtypes	Major genetic abnormalities
Kasumi3	M0	*MECOM*-rearrangement
KG-1a	M1	*NRAS* and *TP53* mutations
HYT-1	M1	No data
Kasumi1	M2	*RUNX1-RUNX1T1* fusion, *KIT, RAD21,* and *TP53* mutations
SKNO1	M2	*RUNX1-RUNX1T1* fusion, *KIT* and *TP53* mutations
Kasumi6	M2	*FLT3*-ITD, *CEBPA* and *TP53* mutations
KO52	M2	*DNMT3A, NRAS*, and *TP53* mutations
HL-60	M2	*CDKN2A, NRAS*, and *TP53* mutations
NB4	M3	*PML-RARA* fusion, *KRAS* and *TP53* mutations
UF-1	M3	*PML-RARA* fusion
ML-2	M4	*KMT2A-MLLT4* fusion, *KRAS* mutation
OCI-AML2	M4	*KMT2A-MLLT4* fusion, *DNMT3A* mutation
OCI-AML3	M4	*DNMT3A, NRAS* and *NPM1* mutations
ME-1	M4	*CBFB-MYH11* fusion, *NRAS* mutation
THP-1	M5	*KMT2A-MLLT3* fusion, *NRAS* and *TP53* mutations
NOMO-1	M5	*KMT2A-MLLT3* fusion, *KRAS* and *TP53* mutations
MV4-11	M5	*KMT2A-AFF1* fusion, *FLT3*-ITD
U937	M5	*PICALM-MLLT10* fusion, *PTPN11, TP53*, and *WT1* mutations
HEL	M6	*JAK2* and *TP53* mutations
KG-1	M6	*NRAS* and *TP53* mutations
M-MOK	M7	No data

Supplementary Table 2

Primer sequences for real-time PCR.

Primers	Sequences
*GAPDH*_Forward	5’-GAAGGTGAAGGTCGGAGTC-3’
*GAPDH*_Reverse	5’-GAAGATGGTGATGGGATTTC-3’
*KLF4*_Forward	5’-GGCACTACCGTAAACACACG -3’
*KLF4*_Reverse	5’-CTGGCAGTGTGGGTCATATC -3’
*DPYSL2A*_Forward	5’-AAGCCCTGCAGAACATCAAC-3’
*DPYSL2A*_Reverse	5’-TTGCTTGATCAACCCATCTTC-3’

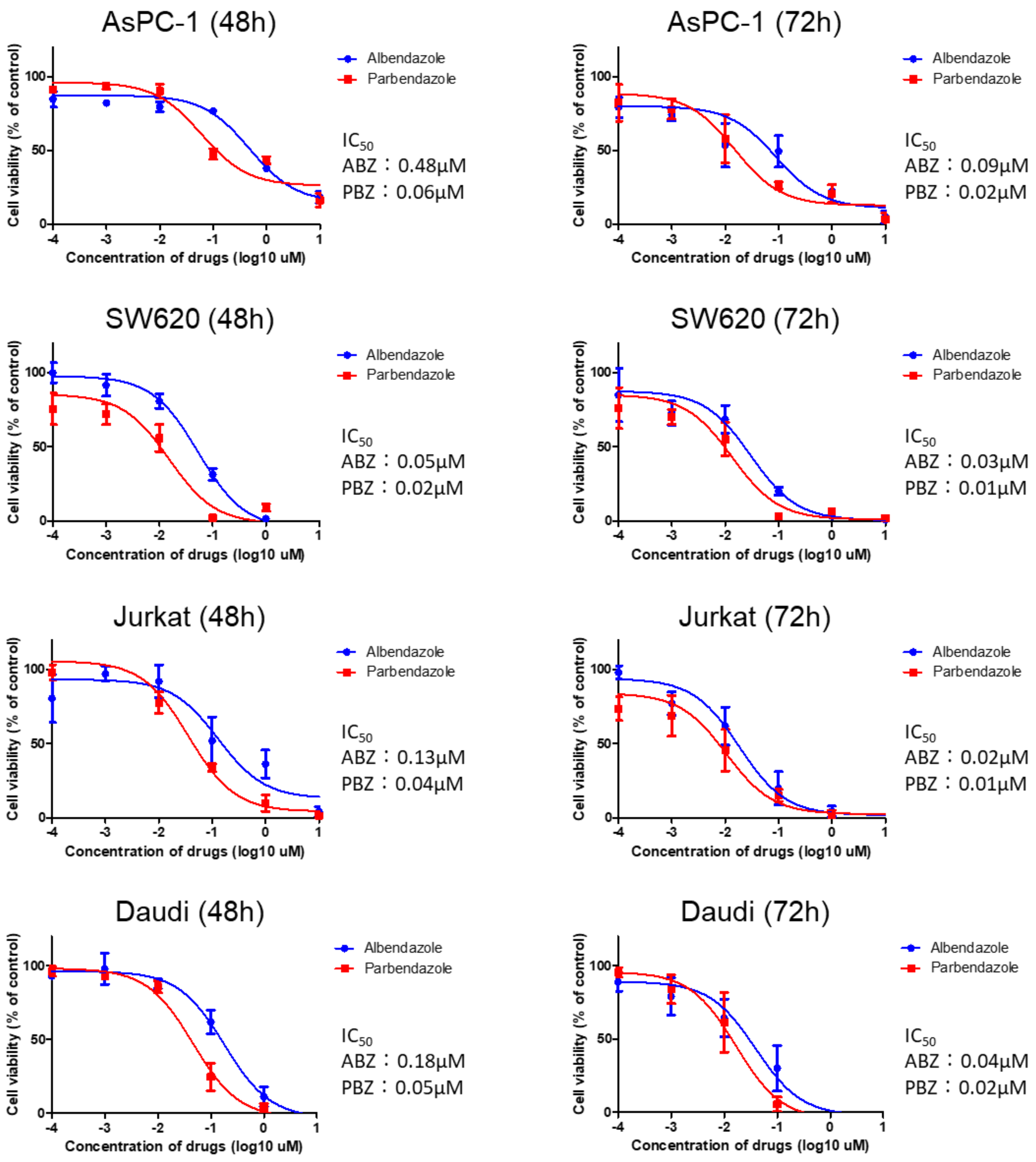

Supplementary Fig. 1

IC_{50} values of Albendazole and Parbendazole for various cancer cell lines.

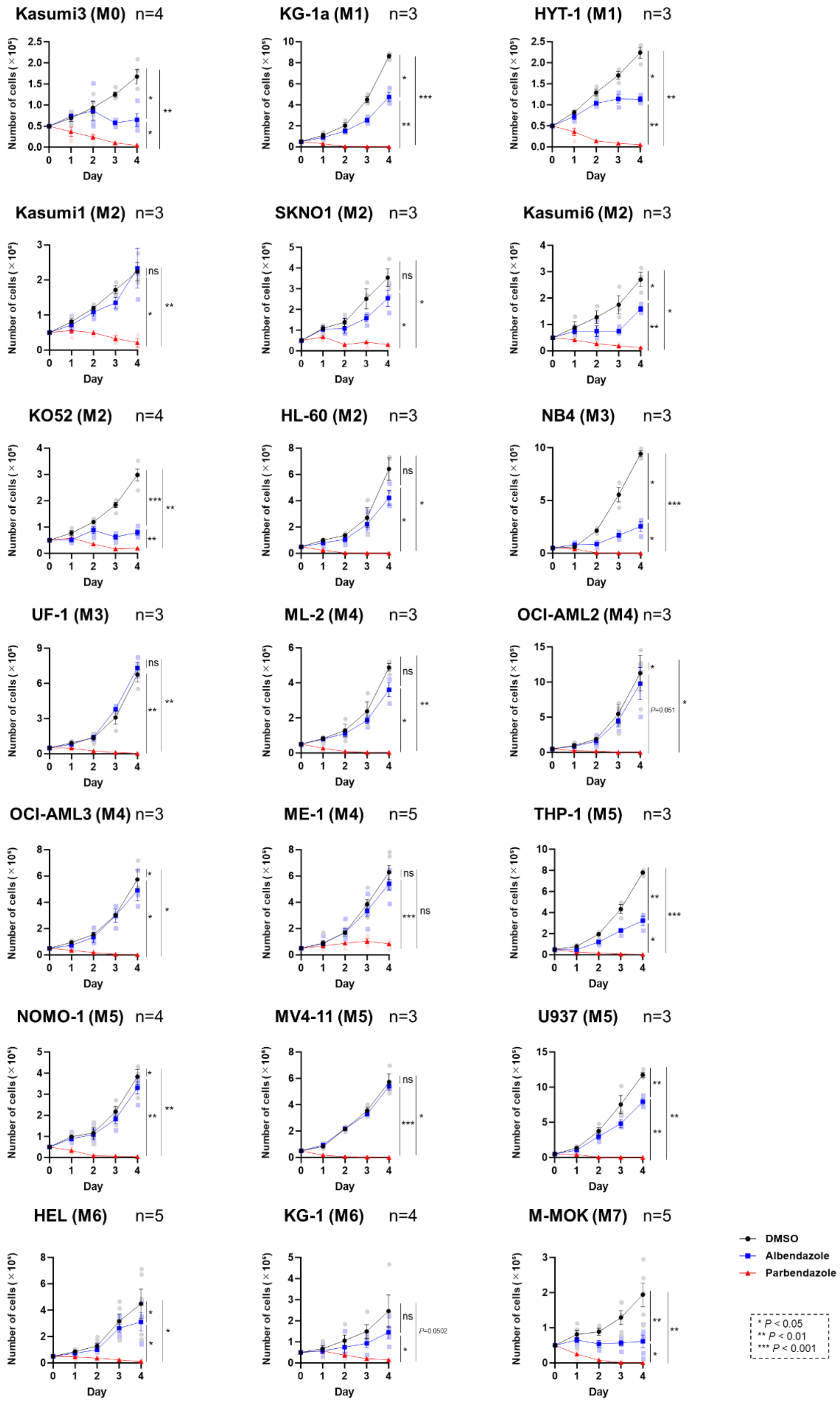

Supplementary Fig. 2

Effects of Albendazole and Parbendazole on the proliferation of AML cell lines.

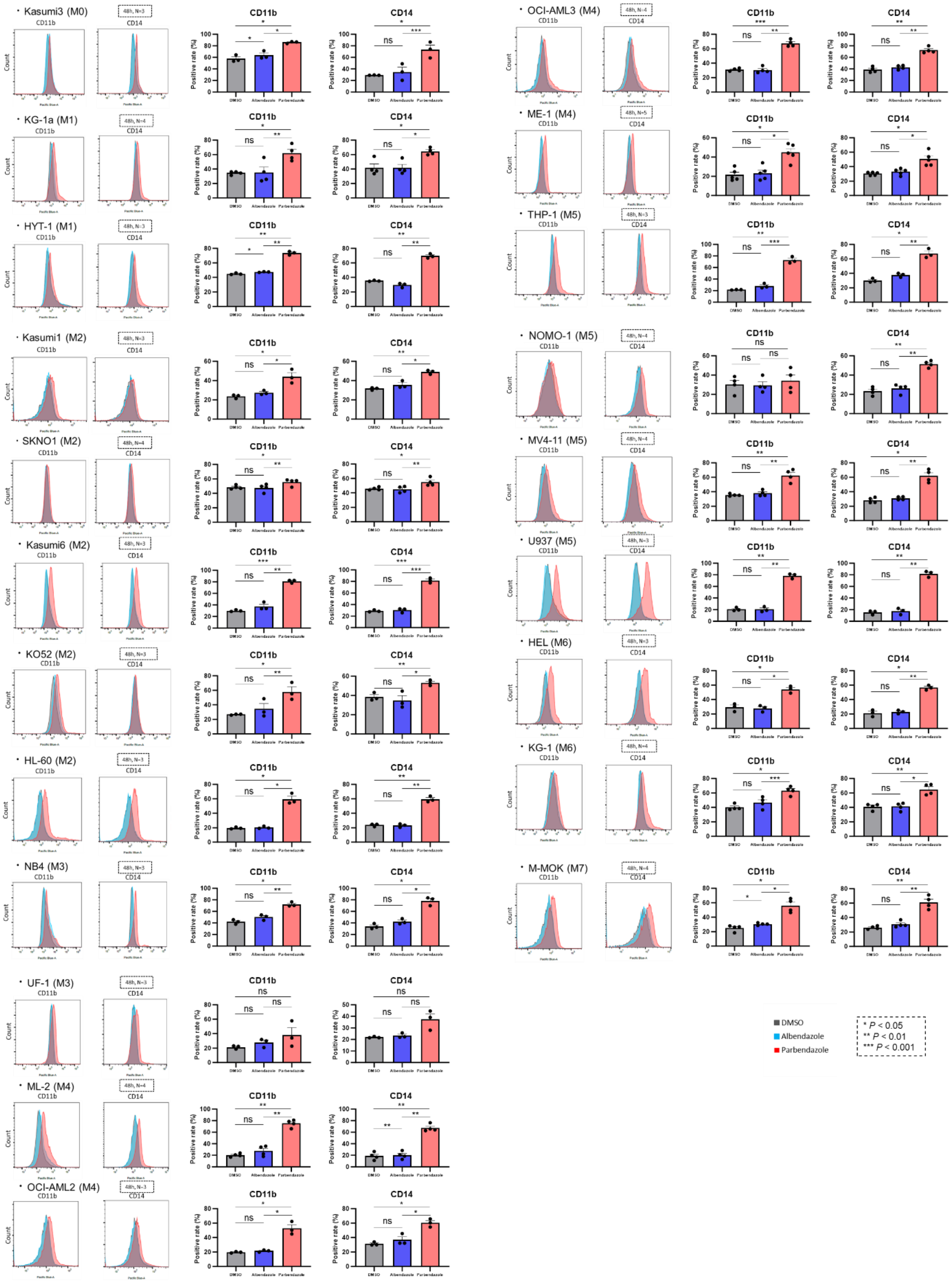

Supplementary Fig. 3

Effects of Albendazole and Parbendazole on the monocyte marker (CD11b, CD14) expression of AML cell lines.

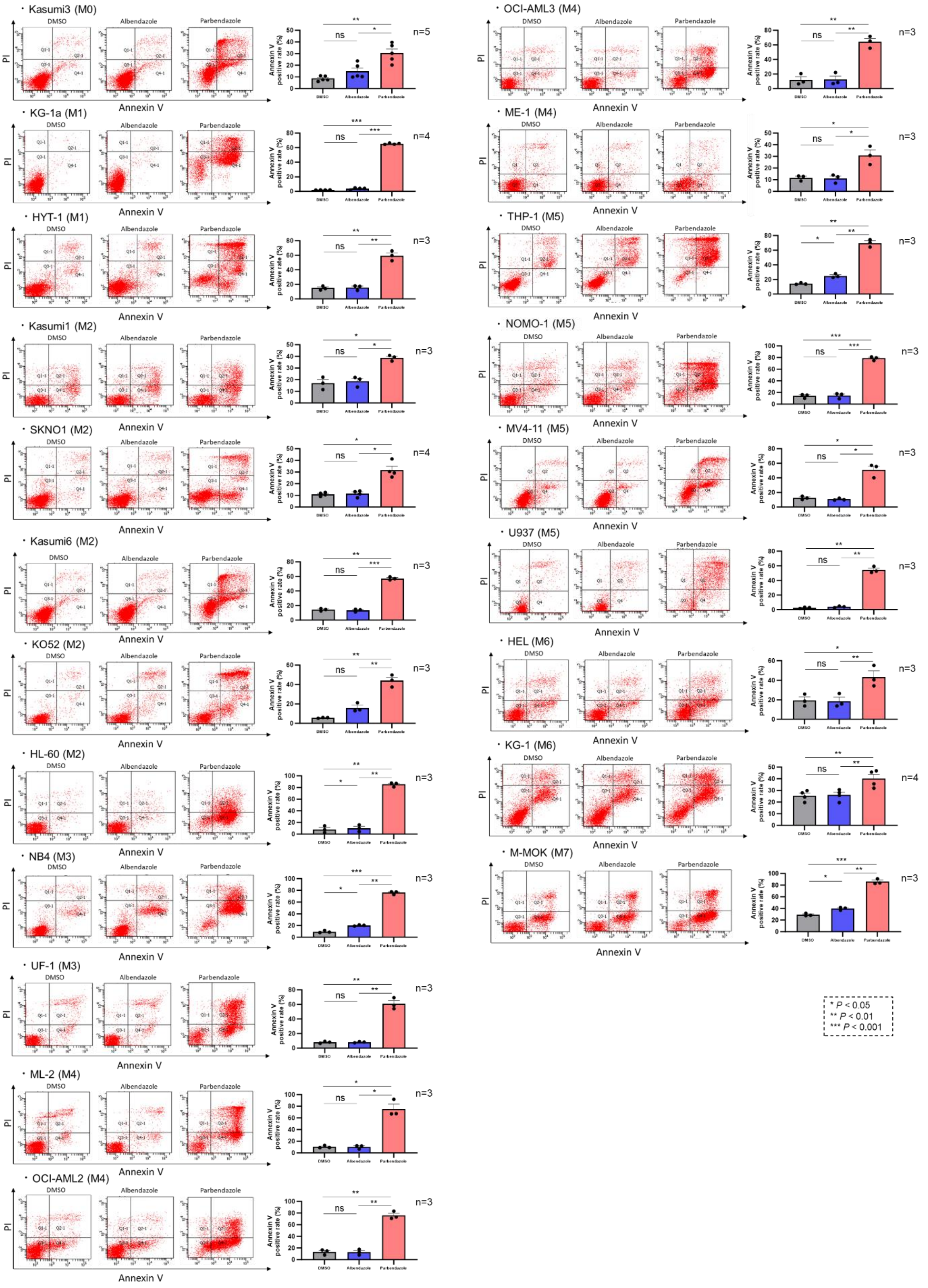

Supplementary Fig. 4

Effects of Albendazole and Parbendazole on the apoptosis of AML cell lines.

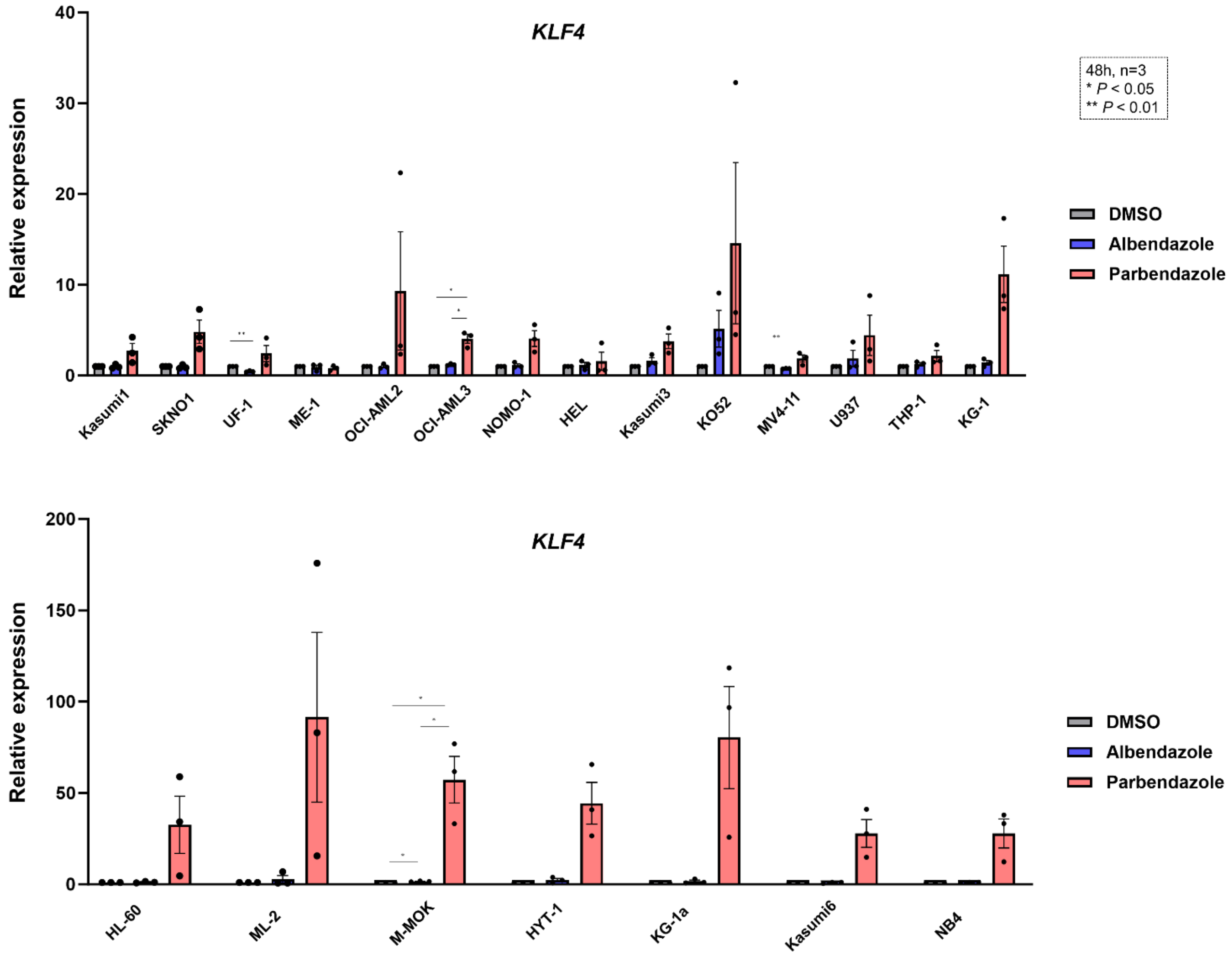

Supplementary Fig. 5

Effects of Albendazole and Parbendazole on the *KLF4* expression of AML cell lines.

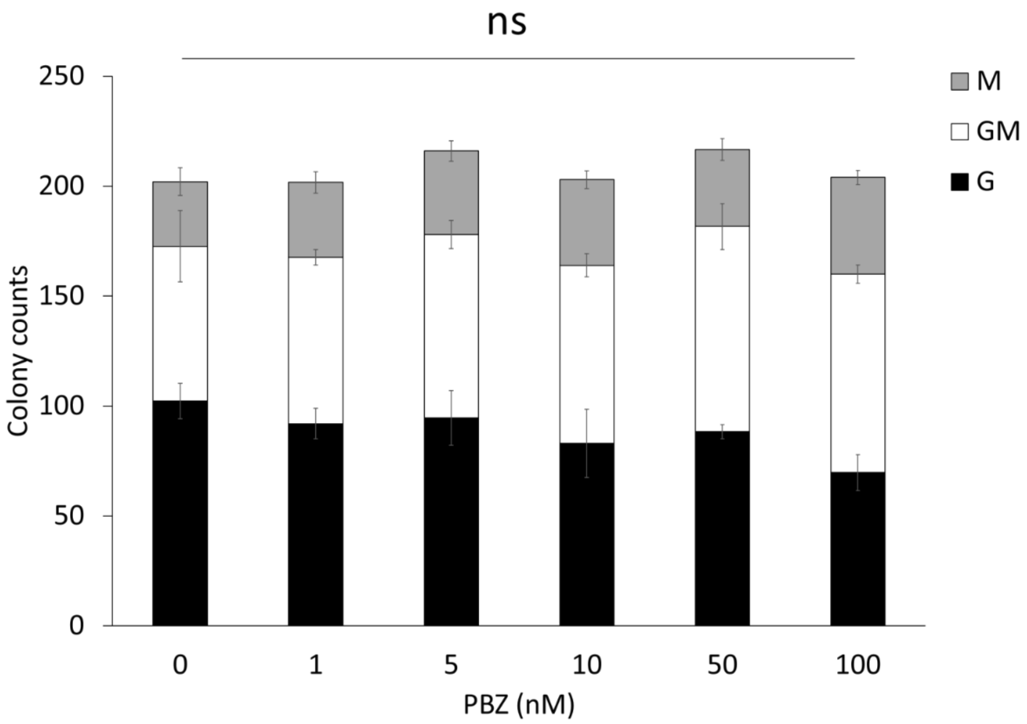

Supplementary Fig. 6

Effects of Parbendazole on the colony-forming capacity of the c-kit+ immature bone marrow cells from wild-type mice.

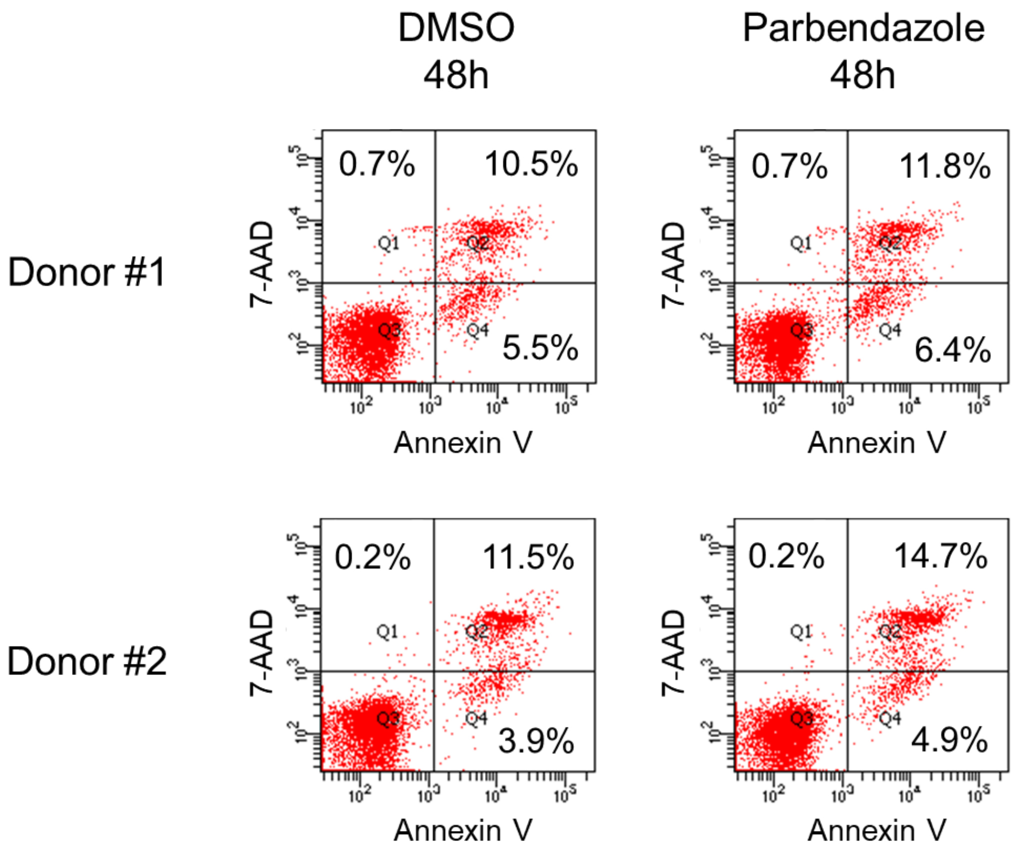

Supplementary Fig. 7

Effects of Parbendazole on apoptosis of the human cord blood CD34+ cells derived from two donors.

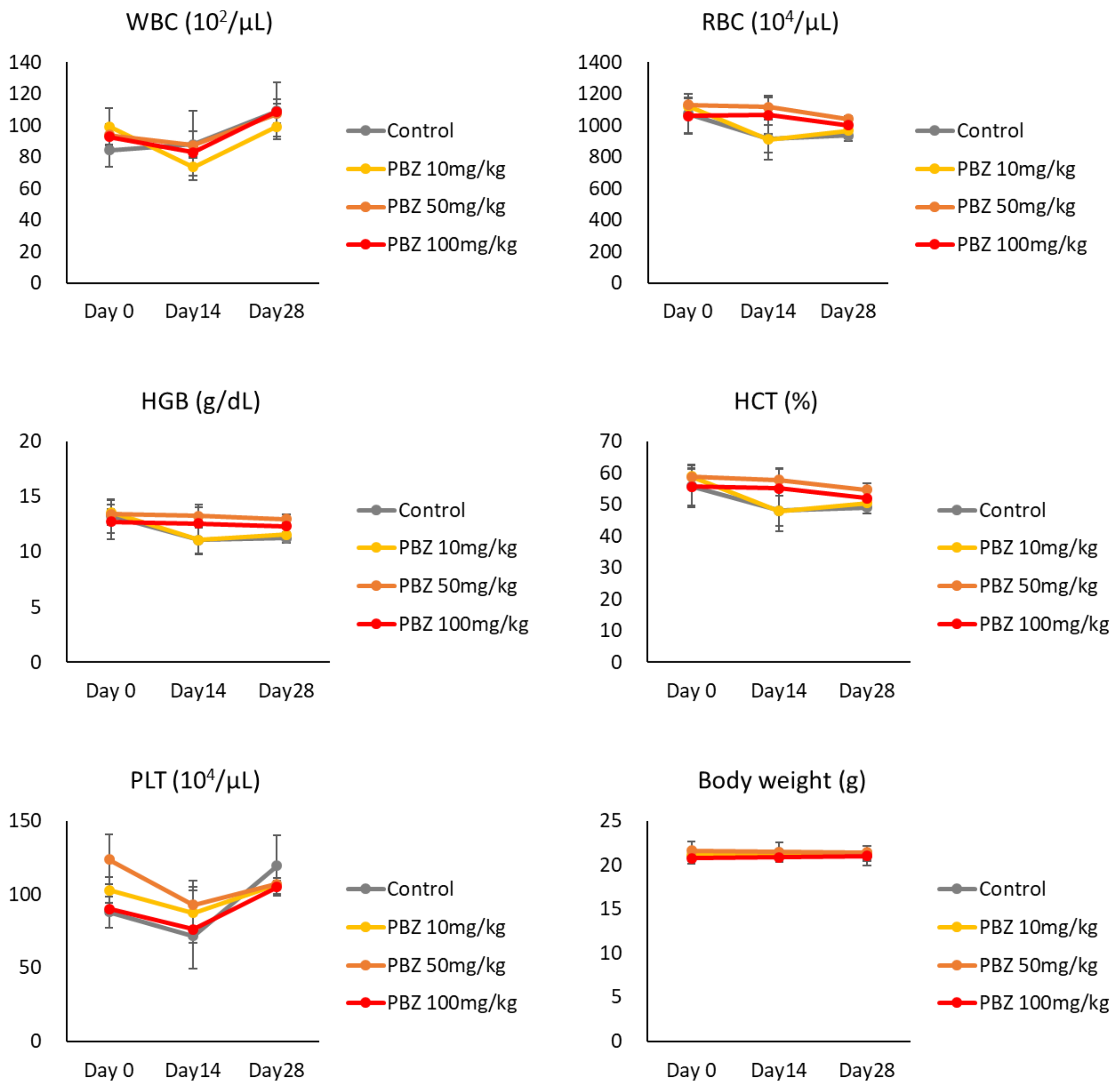

Supplementary Fig. 8

Effects of Parbendazole on blood cell counts and body weight in C57BL/6J mice (n=3 in each group).

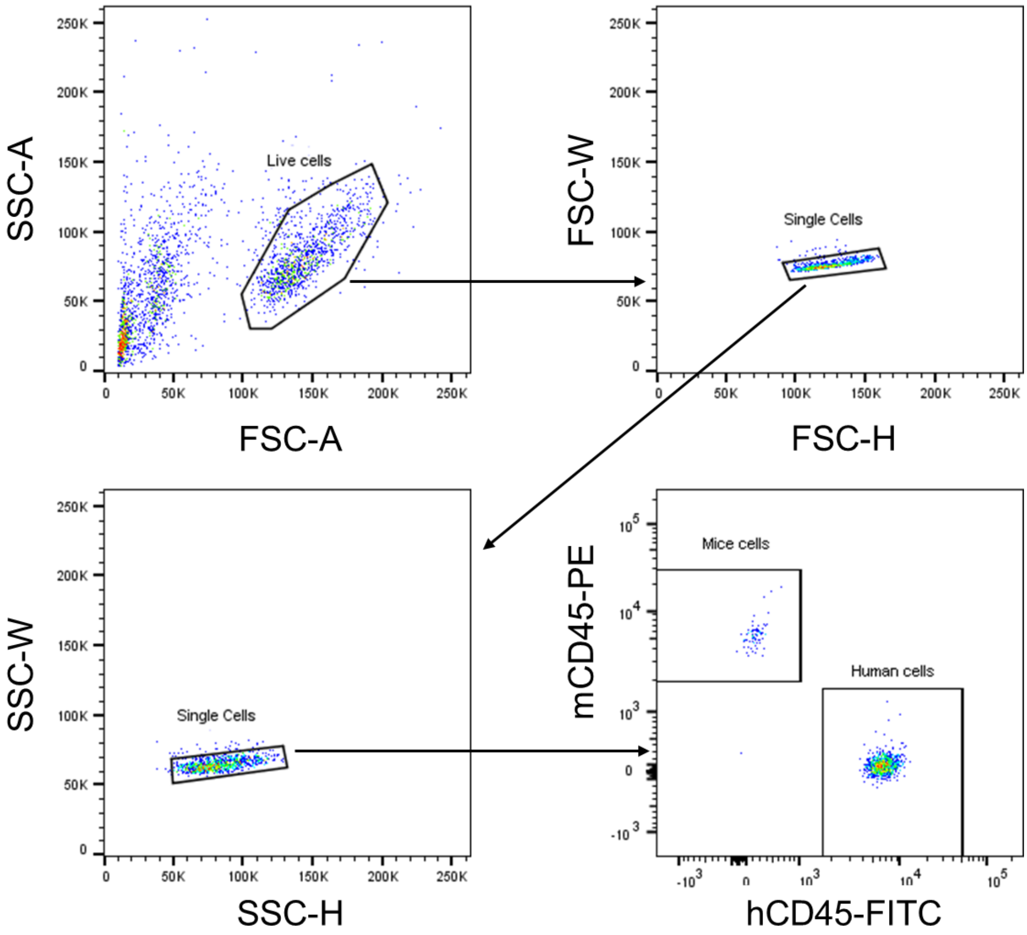

Supplementary Fig. 9

Gating strategy for chimerism analysis.
