# Supplementary material for: Parbendazole as a promising drug for inducing differentiation of acute myeloid leukemia cells with various subtypes
Source: Commun Biol. 2024 Jan 24;7:123. doi: 10.1038/s42003-024-05811-8 (PMC10808455; doi:10.1038/s42003-024-05811-8)
Supplement: Supplementary file 2 — Description of Additional Supplementary Files [file 42003_2024_5811_MOESM2_ESM.pdf]

Description of Additional Supplementary Files

File name: Supplementary Data 1
Description: All source data for the graphs and charts.
