# Supplementary material for: Parbendazole as a promising drug for inducing differentiation of acute myeloid leukemia cells with various subtypes
Source: Commun Biol. 2024 Jan 24;7:123. doi: 10.1038/s42003-024-05811-8 (PMC10808455; doi:10.1038/s42003-024-05811-8)
Supplement: Supplementary file 4 — Reporting Summary [file 42003_2024_5811_MOESM4_ESM.pdf]

Corresponding author(s): Hidemasa Matsuo
Last updated by author(s): Dec 20, 2023

Reporting Summary

Nature Portfolio wishes to improve the reproducibility of the work that we publish. This form provides structure for consistency and transparency in reporting. For further information on Nature Portfolio policies, see our Editorial Policies and the Editorial Policy Checklist.

Statistics

For all statistical analyses, confirm that the following items are present in the figure legend, table legend, main text, or Methods section.

n/a	Confirmed	
☐	☒	The exact sample size (*n*) for each experimental group/condition, given as a discrete number and unit of measurement
☐	☒	A statement on whether measurements were taken from distinct samples or whether the same sample was measured repeatedly
☐	☒	The statistical test(s) used AND whether they are one- or two-sided<br>*Only common tests should be described solely by name; describe more complex techniques in the Methods section.*
☒	☐	A description of all covariates tested
☐	☒	A description of any assumptions or corrections, such as tests of normality and adjustment for multiple comparisons
☐	☒	A full description of the statistical parameters including central tendency (e.g. means) or other basic estimates (e.g. regression coefficient) AND variation (e.g. standard deviation) or associated estimates of uncertainty (e.g. confidence intervals)
☐	☒	For null hypothesis testing, the test statistic (e.g. *F*, *t*, *r*) with confidence intervals, effect sizes, degrees of freedom and *P* value noted<br>*Give P values as exact values whenever suitable.*
☒	☐	For Bayesian analysis, information on the choice of priors and Markov chain Monte Carlo settings
☒	☐	For hierarchical and complex designs, identification of the appropriate level for tests and full reporting of outcomes
☒	☐	Estimates of effect sizes (e.g. Cohen's *d*, Pearson's *r*), indicating how they were calculated

Our web collection on statistics for biologists contains articles on many of the points above.

Software and code

Policy information about availability of computer code

Data collection	Step One Plus™ Real-Time PCR System; Infinite® 200 PRO multimode reader; Countess™ II Automated Cell Counter; BD FACS Canto™ II; BD FACS Aria™ II.
Data analysis	Step One Plus™ Real-Time PCR System; Flow Jo software; GraphPad Prism 5/10 Software; Microsoft Excel.

For manuscripts utilizing custom algorithms or software that are central to the research but not yet described in published literature, software must be made available to editors and reviewers. We strongly encourage code deposition in a community repository (e.g. GitHub). See the Nature Portfolio guidelines for submitting code & software for further information.

Data

Policy information about availability of data

All manuscripts must include a data availability statement. This statement should provide the following information, where applicable:

- Accession codes, unique identifiers, or web links for publicly available datasets
- A description of any restrictions on data availability
- For clinical datasets or third party data, please ensure that the statement adheres to our policy

The main data supporting the findings of this study are available in the manuscript and its supplementary file. All other data are available from the corresponding author upon reasonable request.

Human research participants

Policy information about studies involving human research participants and Sex and Gender in Research.

Reporting on sex and gender	Sex and gender were not considered in this study.
Population characteristics	See above.
Recruitment	Primary AML cells were obtained from the bone marrow of a pediatric AML patient at Kyoto University Hospital after an institutional review board approval (Permit Number: G1030) and providing informed consent.
Ethics oversight	See above. This study was conducted following the Declaration of Helsinki.

Note that full information on the approval of the study protocol must also be provided in the manuscript.

Field-specific reporting

Please select the one below that is the best fit for your research. If you are not sure, read the appropriate sections before making your selection.

☒ Life sciences ☐ Behavioural & social sciences ☐ Ecological, evolutionary & environmental sciences

For a reference copy of the document with all sections, see nature.com/documents/nr-reporting-summary-flat.pdf

Life sciences study design

All studies must disclose on these points even when the disclosure is negative.

Sample size	For in vitro and in vivo studies: n≥3.
Data exclusions	For in vitro and in vivo studies: no data were excluded from the analyses.
Replication	For in vitro studies: all experimental findings were reproduced for at least three times with similar results.
Randomization	All were randomly assigned.
Blinding	Blinding is not relevant to this study because it is not a clinical study that requires group assignment, such as a clinical trial.

Reporting for specific materials, systems and methods

We require information from authors about some types of materials, experimental systems and methods used in many studies. Here, indicate whether each material, system or method listed is relevant to your study. If you are not sure if a list item applies to your research, read the appropriate section before selecting a response.

Materials & experimental systems

n/a	Involved in the study	
☐	☒	Antibodies
☐	☒	Eukaryotic cell lines
☒	☐	Palaeontology and archaeology
☐	☒	Animals and other organisms
☒	☐	Clinical data
☒	☐	Dual use research of concern

Methods

n/a	Involved in the study	
☒	☐	ChIP-seq
☐	☒	Flow cytometry
☒	☐	MRI-based neuroimaging

Antibodies

Antibodies used	BV421-labeled anti-human CD11b (301324; BioLegend, USA); BV421-labeled anti-human CD14 (325628; BioLegend, USA); PE-labeled anti-mouse CD45 (561087; BD Biosciences, USA); FITC-labeled anti-human CD45 (368508; BioLegend, USA); APC Annexin V Apoptosis Detection Kit with PI (640932; BioLegend, USA) or 7-AAD (420403; BioLegend, USA).
Validation	All antibodies used in this study are commercially available and were used for the applications validated by the manufacturers.

Eukaryotic cell lines

Policy information about cell lines and Sex and Gender in Research

Cell line source(s)	HYT-1, THP-1, KG-1a, and M-MOK cells were purchased from RIKEN BioResource Research Center, Japan. Kasumi1, Kasumi3, Kasumi6, SKNO1, HL-60, NOMO-1, Jurkat, and Daudi cells were obtained from the Japanese Collection of Research Bioresources (Japan). KO52, ML-2, OCI-AML2, OCI-AML3, MOLM13, MV4-11, U937, and HEL cells were purchased from Deutsche Sammlung von Mikroorganismen und Zellkulturen GmbH (DSMZ, Germany). ATRA-resistant APL-derived NB4 and UF-1 cells were kindly provided by Dr. Y. Ikeda (Keio University School of Medicine, Japan). AML-derived ME-1 cells were a gift from Dr. PP Liu (National Human Genome Research Institute, National Institutes of Health, USA). AsPC-1 and SW620 cells were purchased from the American Type Culture Collection (ATCC, USA).
Authentication	Cells were authenticated as per the supplier's product sheet, safety data sheet, and certificate of analysis.
Mycoplasma contamination	The cells were tested to be negative for mycoplasma contaminations.
Commonly misidentified lines (See ICLAC register)	No commonly misidentified cell lines were used.

Animals and other research organisms

Policy information about studies involving animals; ARRIVE guidelines recommended for reporting animal research, and Sex and Gender in Research

Laboratory animals	Female C57BL/6J mice were purchased from CLEA Japan, Inc., and male NOG mice were obtained from the Central Institute for Experimental Animals, Inc.
Wild animals	N/A
Reporting on sex	Our findings are not affected by the sex of the animals.
Field-collected samples	N/A
Ethics oversight	All procedures performed in this study were approved by the Kyoto University Animal Experimentation Committee (Permit Number: Med Kyo 22530).

Note that full information on the approval of the study protocol must also be provided in the manuscript.

Flow Cytometry

Plots

Confirm that:

☒ The axis labels state the marker and fluorochrome used (e.g. CD4-FITC).

☒ The axis scales are clearly visible. Include numbers along axes only for bottom left plot of group (a 'group' is an analysis of identical markers).

☒ All plots are contour plots with outliers or pseudocolor plots.

☒ A numerical value for number of cells or percentage (with statistics) is provided.

Methodology

Sample preparation	Monocytic AML cell differentiation was assessed using brilliant violet 421 (BV421)-labeled anti-human CD11b (301324; BioLegend, USA) and CD14 (325628; BioLegend, USA) antibodies. The living cell population was gated in a forward scatter/side scatter dot plot; then, CD11b, and CD14 expressions were analyzed. Apoptotic cells were determined using the APC Annexin V Apoptosis Detection Kit with PI (640932; BioLegend, USA) or 7-AAD (420403; BioLegend, USA). For chimerism analysis, bone marrow cells were collected by bone marrow aspiration and reacted with PE-labeled anti-mouse CD45 (561087; BD Biosciences, USA) and FITC-labeled anti-human CD45 (368508; BioLegend, USA) antibodies on ice for 30 min in the dark. The chimerism percentage in the bone marrow of mice was evaluated using the fact that mouse-derived cells are mCD45-positive and leukemia cells are hCD45-positive. The gating strategy is provided in the Supplementary Fig. 9. Measurements/cell sorting were performed using a BD FACS Canto™ II or BD FACS Aria™ II (BD Biosciences, USA). The Flow Jo software (BD Biosciences, USA) was used for data analysis. To determine the CD11b/CD14 positivity rate for each cells, a positivity threshold was determined using the histogram of the DMSO-treated samples (control). The CD11b/CD14 positivity rate in ABZ/PBZ treated samples was then examined using the threshold. The results are presented as the mean ± SEM of the values obtained from at least three independent experiments. For colony-forming assay, c-kit+ primary bone marrow cells of the mouse were isolated from C57BL/6J mice using APC anti-mouse c-kit antibody (105812; BioLegend, USA) and BD FACS Aria™ II (BD Biosciences, USA).
Instrument	BD FACS Canto™ II and BD FACS Aria™ II (BD Biosciences, USA)

Software	Flow Jo software (BD Biosciences, USA)
Cell population abundance	For targeting cell population, at least 10,000 events were recorded.<br>For cell sorting, the cell purity was > 90%.
Gating strategy	After removing debris using FSC and SSC gates, doublet was excluded by plotting Height and Width in FSC and SSC. See methods and figures for details.

☒ Tick this box to confirm that a figure exemplifying the gating strategy is provided in the Supplementary Information.
